# Inhibition of Metastatic Potential in Breast Carcinoma *In Vivo* and *In Vitro* through Targeting VEGFRs and FGFRs

**DOI:** 10.1155/2013/718380

**Published:** 2013-06-03

**Authors:** Ming-Hsien Chien, Liang-Ming Lee, Michael Hsiao, Lin-Hung Wei, Chih-Hau Chen, Tsung-Ching Lai, Kuo-Tai Hua, Min-Wei Chen, Chung-Ming Sun, Min-Liang Kuo

**Affiliations:** ^1^Graduate Institute of Clinical Medicine, College of Medicine, Taipei Medical University, Taipei 11031, Taiwan; ^2^Wan Fang Hospital, Taipei Medical University, Taipei 116, Taiwan; ^3^Department of Urology, Wan Fang Hospital, Taipei Medical University, Taipei 116, Taiwan; ^4^The Genomics Research Center, Academia Sinica, Taipei 115, Taiwan; ^5^Department of Oncology, National Taiwan University Hospital, Taipei 10041, Taiwan; ^6^Department of Applied Chemistry, National Chiao Tung University, No. 1001 Ta Hsueh Road, Hsinchu 300-10, Taiwan; ^7^Graduate Institute of Toxicology, College of Medicine, National Taiwan University, Taipei 100, Taiwan; ^8^Graduate Institute of Biomedical Sciences, College of Life Science, National Taiwan University, No. 1, Section 4, Roosevelt Road, Taipei 106, Taiwan

## Abstract

Angiogenesis and lymphangiogenesis are considered to play key roles in tumor metastasis. Targeting receptor tyrosine kinases essentially involved in the angiogenesis and lymphangiogenesis would theoretically prevent cancer metastasis. However, the optimal multikinase inhibitor for metastasis suppression has yet to be developed. In this study, we evaluated the effect of NSTPBP 0100194-A (194-A), a multikinase inhibitor of vascular endothelial growth factor receptors (VEGFRs)/fibroblast growth factor receptors (FGFRs), on lymphangiogenesis and angiogenesis in a mammary fat pad xenograft model of the highly invasive breast cancer cell line 4T1-Luc^+^. We investigated the biologic effect of 194-A on various invasive breast cancer cell lines as well as endothelial and lymphatic endothelial cells. Intriguingly, we found that 194-A drastically reduced the formation of lung, liver, and lymph node metastasis of 4T1-Luc^+^ and decreased primary tumor growth. This was associated with significant reductions in intratumoral lymphatic vessel length (LVL) and microvessel density (MVD). 194-A blocked VEGFRs mediated signaling on both endothelial and lymphatic endothelial cells. Moreover, 194-A significantly inhibited the invasive capacity induced by VEGF-C or FGF-2 *in vitro* in both 4T1 and MDA-MB231 cells. In conclusion, these experimental results demonstrate that simultaneous inhibition of VEGFRs/FGFRs kinases may be a promising strategy to prevent breast cancer metastasis.

## 1. Introduction

Tissue invasion and metastasis, which cause 90% of cancer deaths, are common features during the development of most types of human cancer. The distant settlements of tumor cells can be, in general, classified into hematogenous metastasis and lymphogenous metastasis. Although invasion and metastasis are exceedingly complex processes, recent advances in understanding the molecular mechanisms involved in angiogenesis and lymphangiogenesis have provided opportunities to develop new treatments to prevent metastasis. 

 Tumors express various angiogenic and lymphangiogenic factors. VEGF family, among all, is perhaps the most important one. VEGF-A, the founding member of the family, has emerged as the key mediator of neovascularization in cancer [1]. The biological functions of the VEGFs are mediated by a family of cognate protein tyrosine kinase receptors (VEGFRs) [2–4]. VEGF-A binds to VEGFR-2 and VEGFR-1; VEGF-C and VEGF-D bind VEGFR-2 and VEGFR-3; PLGF and VEGF-B bind only to VEGFR-1; VEGF-E binds only to VEGFR-2. Signaling through VEGFR-2 and VEGFR-3 is crucial in the promotion of angiogenesis and lymphangiogenesis, respectively [5, 6]. In addition to the expression on endothelial cells/lymphatic endothelial cells, VEGFR-2/VEGFR-3 has been shown to be expressed in a variety of human malignancies, including breast carcinoma [7, 8]. 

Much research has determined that the VEGF-A/VEGFR-2 axis in cancer cells can promote growth of cancer cells [9], while the VEGF-C/VEGFR-3 axis enhances mobility of cancer cells and contributes to the promotion of metastasis in animals [10]. Given a significant role of VEGFR-2/VEGFR-3 in tumor development and progression, inhibition of both VEGF-A/VEGFR-2 and VEGF-C/VEGFR-3 signals has shown promising results in suppressing tumor progression and metastasis in preclinical studies [11].

Overexpression of fibroblast growth factor receptor (FGFR) tyrosine kinases has been found in human breast cancers and has been associated with poor patient prognosis [12, 13]. There are four FGFR genes (*FGFR1*–*FGFR4*) that encode receptors consisting of three extracellular immunoglobulin domains, a single-pass transmembrane domain, and a cytoplasmic tyrosine kinase domain [14]. In breast carcinoma, amplification and overexpression of FGFRs, including FGFR-1 (20%), FGFR-2 (12%), and FGFR-4 (30%), have been observed [15–17]. These FGFRs mediate signaling from their high-affinity ligands, fibroblast growth factors (FGFs) [18]. The FGFs/FGFRs signaling interferes with many cellular functions, such as cell proliferation, transformation, and angiogenesis [19]. In particular, recent advances have shown that FGFRs activity is linked to tumor growth, epithelial-mesenchymal transition, and distant metastasis and thus contributes to tumor progression [20–22]. Also, targeting FGFRs signaling has been shown to suppress tumor outgrowth and metastasis in pre-clinical models [23]. Therefore, blocking VEGFRs/FGFRs activities may be of clinical benefit in the management of patients with highly metastatic breast cancer. 

 We have recently discovered a low molecular weight synthetic receptor tyrosine kinase inhibitor with the 2H-indazole core [24]. We identified NSTPBP 0100194-A (194-A) as a compound with particularly strong inhibitory potency against VEGFR-3 and VEGFR-2 kinase activity. In addition, 194-A showed similar potency against FGFR-1, FGFR-2, and FGFR-4, but was largely inactive against other tyrosine kinases. The kinase inhibitory signature of 194-A prompted us to evaluate this compound as a therapeutic for VEGFRs/FGFRs-dependent malignancies. In this study, we determined the effect of 194-A on both angiogenesis and lymphangiogenesis using a 4T1 mammary fat pad model and found that inhibition of VEGFRs/FGFRs dramatically suppressed tumor metastasis to regional lymph nodes and distant organs, via angiogenic and lymphangiogenic inhibition as well as suppressing the metastatic potential of tumor cells. 

## 2. Materials and Methods

### 2.1. Cell Lines

Human umbilical vein endothelial cells (HUVECs) and lymphatic endothelial cells (LECs) were purchased from PromoCell (Heidelberg, Germany). The high invasive breast cancer cell lines, 4T1 and MDA-MB231, were purchased from American Type Culture Collection (Manassas, VA, USA). These cells were cultured according to the vendor's guidelines. 4T1 cells were engineered to express the firefly luciferase protein for detection *in vivo* using Xenogen IVIS-100 imaging system. The luciferase positive population of 4T1 cells was selected in gentamicin (G418; Life Technologies). Bioluminescent, antibiotic resistant, and single-cell clones were amplified in culture and characterized for stable luminescence *in vitro, *and tumorigenic potential monitored *in vivo*.

### 2.2. Kinase Inhibitor

NSTPBP 0100194-A (194-A), 1-(2-cyclohexenylethyl)-2-(2-(3,3-diphenylpropyl)-2H-indazole-6-yl)-1H-benzo[d]imidazole-5-carboxylic acid (see Supplementary Figure 1 in Supplementary Material available online at http://dx.doi.org/10.1155/2013/718380) was provided by Dr. Chung-Ming Sun's laboratory at the Department of Applied Chemistry (National Chiao Tung University, Hsinchu, Taiwan). The synthetic routes were described elsewhere [24] and the kinase inhibitory profile was shown in Supplementary Table 1. For *in vitro* experiments, 194-A was dissolved in DMSO. For *in vivo* experiments, 194-A was prepared in a microemulsion containing 2 mg 194-A, 8.3 mg tricaprin, 50 mg Tween 80, and 20 mg propylene glycol in 1 mL PBS buffer. 

### 2.3. Antibodies and Reagents

 VEGF-C and VEGF-A_165_ were purchased from R&D Systems. The following primary antibodies were used: VEGFR-2, proliferating cell nuclear antigen (PCNA) (Upstate, Lake Placid, NY, USA); p-tyr1054 VEGFR-2 (Millipore); lymphatic vessel endothelial receptor 1 (LYVE-1) (R&D Systems); phosphorylated tyrosine (PY-99), VEGFR-3, phosphorylated extracellular signal-regulated kinase 1/2 (ERK1/2), ERK1/2, phosphorylated Akt, Akt, CD31 (Santa Cruz Biotechnology). Biotin-labeled donkey anti-goat IgG and TRITC-labeled donkey anti-goat IgG secondary antibody were purchased from Santa Cruz Biotechnology. 4′,6-Diamidino-2-phenylindole dihydrochloride (DAPI) was obtained from Sigma-Aldrich. Sunitinib and sorafenib were purchased from Pfizer and Bayer, respectively. 

### 2.4. Immunoprecipitation and Western Blot

Protein lysates were prepared as previously described [25]. Western blotting was performed with primary antibodies for p-tyr1054 VEGFR-2, VEGFR-2, p-ERK1/2, ERK1/2, p-Akt, and Akt, as noted. For immunoprecipitation, protein lysates were incubated with VEGFR-3 antibody immobilized onto protein A-Sepharose (Sigma-Aldrich) for 1 h at 4°C with gentle rotation.

### 2.5. Endothelial Cell Proliferation

5 × 10^3^ HUVECs or LECs were seeded in collagen-coated 96-well plates and allowed to attach overnight. The medium was replaced with serum-free medium containing 194-A or DMSO with 100 ng/mL VEGF-A or 500 ng/mL VEGF-C for 12 h. Cell proliferation was performed by MTS assay (Promega). Data were collected from three replicates.

### 2.6. Endothelial Cell Migration

Assessment of endothelial cell migratory activity was performed as described [26]. 3 × 10^4^ HUVECs or LECs were suspended in serum-free media and seeded in the top chamber of a cell culture insert (Costar, Cambridge, MA, USA) after treatment with DMSO or 194-A for 30 min. The insert was placed in a 24-well plate containing serum-free medium with control protein, VEGF-A (100 ng/mL) or VEGF-C (500 ng/mL); the cells were incubated for 24 h and migrating cells were stained with crystal violet (Sigma, St Louis, MI, USA). The migratory activity was calculated as a percentage of migratory cells in the test samples versus control.

### 2.7. Invasion Assay

Invasion assays were done using modified Boyden chambers with Matrigel (30 *μ*g, Collaborative Biomedical, Becton Dickinson Labware, San Jose, CA, USA) coated filter inserts for 24-well plates. Cells (1 × 10^5^) were pretreated with DMSO or 194-A for 30 min and plated into 100 *μ*L of low serum (1% FBS) RPMI or DMEM in the top chamber. The insert was placed in a 24-well plate containing low serum medium with control protein, FGF-2 (20 ng/mL) or VEGF-C (100 ng/mL) for 24 hr. The cells that invaded through the Matrigel and attached to the lower surface of the filter were stained with crystal violet and calculated.

### 2.8. *In Vitro* Cytotoxicity Assay

 4T-1 cells were plated in 96-well microtiter plates and treated with various concentrations (0, 1, 3, and 10 *μ*M) of 194-A for 24 h, and cell viabilities were assessed using the MTS (Promega Corporation, Madison, WI, USA) assay. The absorbance (*A*) was read at 490 nm using an ELISA reader (MQX200; BioTek Instruments, Winooski, VT, USA).

### 2.9. 4T1 Tumor Model *In Vivo*

Female BALB/c mice were orthotopically injected with 2.5 × 10^5^ 4T1-Luc^+^ cells, suspended in PBS, into the right fat pad. Tumors were measured every three days according to (tumor size = length × width^2^  × 0.52). Mice were given 194-A, sorafenib (in cremophor EL/ethanol), or sunitinib (in citrate-buffer solution) as oral administrations of 50 mg/kg/day or other dosage, as noted. Treatment was initiated after tumors reached 75 mm^3^ and lasted until the endpoint of the experiment. Mice were imaged once a week, by injecting 150 mg/kg luciferin i.p. and imaging the tumors using bioluminescence technology (Xenogen IVIS-100 imaging system). For primary tumors, the exposure time ranged from 5 seconds to 1 minute depending on the size of the tumor. For detection of metastatic tumor nodules, exposure time was extended to 5 minutes. To exclude treatment related toxicity, mice were weighted every two days. Lymph node, distal organs (lung and liver) metastasis, and primary tumor weights were excised at the end of the experiment. All experiments were repeated at least twice with a minimum of 5 mice per group.

### 2.10. Tumor Vascularity Detection *In Vivo*

Tumor vascularity was monitored by using non-contrast-enhanced flow-sensitive ultrasound (Vevo 770 micro-ultrasound system). Non-contrast-enhanced flow-sensitive ultrasound predominantly visualized the vessel networks on the tumor margins, some of which branched toward the tumor center. Tumor vascularity was quantified in power Doppler images by computing the color pixel density, which is equal to the percentage of image voxels within a region of interest that exhibits detectable flow.

### 2.11. Pharmacokinetics of p.o. 194-A Administration in Mice

BALB/C mice received a single dose of 50 mg/kg 194-A by oral gavage, and plasma samples were collected at different time points. The plasma samples were prepared for ultrahigh performance liquid chromatography coupled to tandem mass spectrometry (UPLC/MS/MS) analysis, by protein precipitation with two volumes of acetonitrile (100 *μ*L) per 50 *μ*L plasma sample. Pharmacokinetic parameters were determined by MassLynx 4.1 software.

### 2.12. Immunohistochemistry

Tumor tissues were processed for either paraffin or OCT sections as previously described [27]. CD31 and PCNA staining was detected using streptavidin-biotin peroxidase complex method by DAB Peroxidase Substrate Kit (SK-4100; Vector Laboratories). Detection of LYVE-1 was performed using TRITC-conjugated donkey anti-goat IgG secondary antibody under a Zeiss Axioskop fluorescence microscope. Microvessel density (MVD) and lymphatic vessel length (LVL) were quantified for each 200x field using ProImage software. For each tumor section, 3-4 fields were counted. The number of PCNA-positive cells, among at least 500 cells per field, was counted and expressed as percentage values.

### 2.13. *In Vivo* Cell Death Analysis

DeadEnd Fluorometric Terminal Deoxynucleotidyl Transferase-Mediated Nick-End Labeling System (Promega, Madison, WI) was used to evaluate the cell death in sections of 4T1 tumors obtained from control and test compounds-treated animals, according to the manufacturer's instructions.

### 2.14. Statistical Analysis

Differences between the means of unpaired samples were evaluated by the Student's *t*-test and differences in the median values between the two groups were evaluated by Wilcoxon rank-sum test using the SigmaPlot and SigmaStat programs. *P* < 0.05 was considered statistically significant. All statistical tests were of two sided.

## 3. Results

### 3.1. The *In Vivo* and *In Vitro* Antitumor Activity of 194-A in a Metastasis-Specific Mouse Mammary Carcinoma 4T1

**Figure 1:**
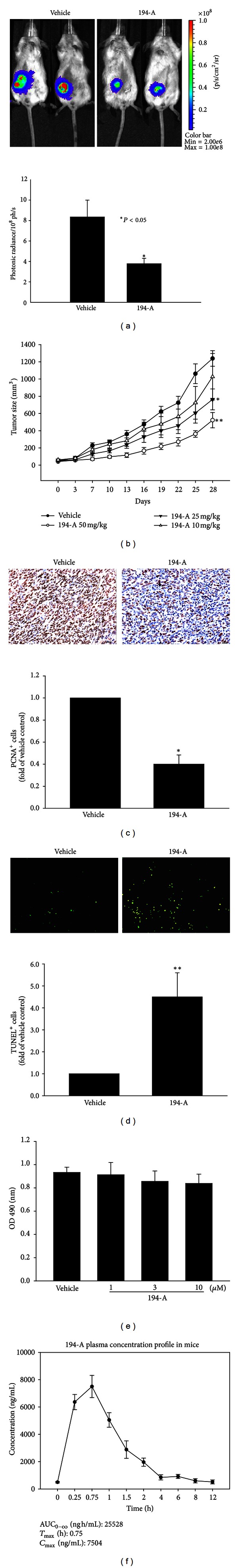
The *in vivo* and *in vitro* antitumor activity of 194-A in the 4T1 orthotopic graft model. (a) and (b) Tumor growth inhibition after treatment with 194-A. 4T1-Luc^+^ (5 × 10^5^ cells/mouse) were orthotopically implanted into the fat pad of male BALB/c mice. The tumor growth inhibition by p.o. administration of 194-A at different dosages (10~50 mg/kg/day) was monitored based on emitted bioluminescence detection (a) or external measurement using a caliper (b). (c) and (d) 194-A induced inhibition of proliferation and induction of apoptosis in 4T1 orthotopic grafts. Proliferation index and apoptotic index were determined by PCNA immunohistochemical staining (c) and TUNEL assay (d), respectively, after 10-day 194-A treatment. PCNA-positive or TUNEL-positive cells were counted in five 200x fields per 4T1 tumor section (*n* = 6). The average number of PCNA-positive or TUNEL-positive cells in each section was normalized to vehicle control. (e) 194-A (1~10 *μ*M) have no significant effects on 4T1 cells proliferation as measured by MTS analysis.* Lines* or *columns*, mean (*n* = 6); *bars*, SE. *P < 0.05; **P < 0.01 as compared to the vehicle control group. Scale bar, 20 *μ*m. (f) 194-A plasma concentration in mice after a single p.o. dose of 50 mg/kg (*n* = 3).

To evaluate the antitumor activity of 194-A on primary tumor growth and metastasis, a mouse mammary carcinoma cell line, 4T1, was used. 4T1 is a highly metastatic tumor cell that can metastasize to the lung, liver, and lymph nodes while the primary tumor is growing *in situ* [28]. 194-A was administered p.o. and evaluated in an orthotopic graft model. 4T1-Luc^+^ cells were inoculated into the mammary fat pad and allowed to establish for 9 days before initiation of treatment. 4T1-Luc^+^ orthotopic graft mice were treated with daily administrations of different dosages of 194-A (10~50 mg/kg) or vehicle control using oral gavage.  Figure 1(a) showed the inhibitory potency of 194-A (50 mg/kg) on tumor growth after 10 days of treatment by photon emissions detection *in vivo*. The mean tumor volume from caliper measurement showed that treatment with 194-A resulted in a dose-dependent inhibition of tumor growth (Figure 1(b)). In 194-A-treated mice receiving 25 or 50 mg/kg daily, the mean tumor volume on day 30 was inhibited by 38% (*P* < 0.05) and 55% (*P* < 0.01), respectively, relative to the vehicle-treated 4T1 tumors (Figure 1(b)). Next, we examined the effects of 194-A on cell proliferation and apoptosis within the 4T1 tumors after 10 days of treatment. The immunohistochemical analysis of cell proliferation was performed using PCNA staining. The mean number of PCNA positive tumor cells was reduced with 60% after treatment with 194-A compared to control mice (Figure 1(c)). Additionally, the number of TUNEL positive cells was increased 4.3-fold in the 194-A treated group compared to the control group (Figure 1(d)). However, 194-A, at a concentration up to 10 *μ*M for 24 h treatment, had no significant effect on 4T1 cells growth *in vitro *(Figure 1(e)). These experimental results clearly verify that 194-A can suppress *in vivo* tumor growth of 4T1 cells without significantly altering their *in vitro* growth rate. Pharmacokinetic studies revealed a maximal plasma concentration (*C*_max⁡_)  ~7500 ng/mL at 0.75 hour, while the plasma levels were below 500 ng/mL at 12 hours after administration of 50 mg/kg 194-A. The p.o. bioavailability of 194-A in BALB/c mice was ~55%. The rapid reduction in 194-A levels implies that 194-A rapidly metabolize to another metabolite, but which metabolite exhibited antitumor activity *in vivo* must be further explored (Figure 1(f)).

### 3.2. The Antiangiogenesis Efficacy of 194-A in the 4T1 Tumor Model

**Figure 2:**
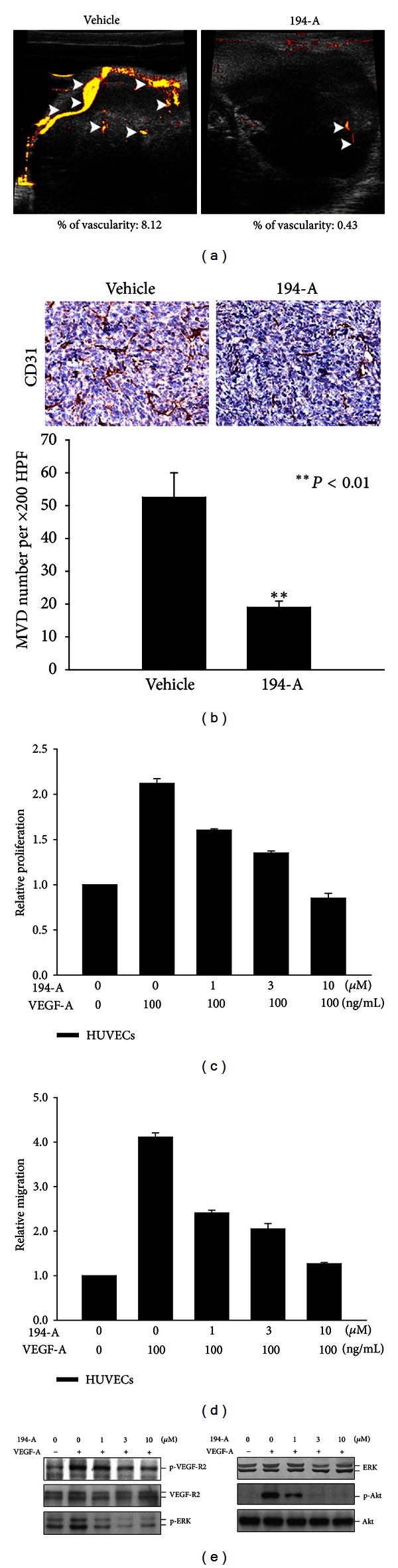
The antiangiogenesis efficacy of 194-A in the 4T1 orthotopic graft model. (a) Tumor vascularity was monitored after 10 days of 194-A (50 mg/kg) p.o. treatment, by using non-contrast-enhanced flow-sensitive ultrasound (Vevo 770 micro-ultrasound system). (b) Immunohistochemical staining of 4T1 tumor sections with CD31 antibody, counterstained with hematoxylin. Vessel density per 200x field was assessed from 3 to 4 fields per tumor section.* Columns*, mean (*n* = 6); *bars*, SE. **P < 0.01 as compared to the vehicle control group. Scale bar, 20 *μ*m. (c) Relative proliferation of HUVECs grown in serum-free media supplemented with 100 ng/mL of VEGF-A as indicated. Proliferation was reduced in a dose-dependent manner in response to 194-A treatment. Mean values of three replicates, normalized to the untreated controls; *bars*, SE. (d) Treatment with increasing concentrations of 194-A reduced VEGF-A-induced migration in HUVECs. The number of migrating cells was normalized to DMSO control and values are displayed as mean values from three independent experiments. (e) 194-A inhibited VEGF-A-induced activation of VEGFR-2 and its common downstream signaling molecules. Serum-starved HUVECs were pretreated with 194-A for 30 min and then stimulated with 100 ng/mL VEGF-A for 10 min. Lysates were resolved in SDS-PAGE and probed with specific antibodies against p-Tyr1054 VEGFR-2, VEGFR-2, p-ERK1/2, ERK1/2 p-Akt, and Akt.

194-A exhibited significant activity against VEGFR-2 (Supplementary Table 1), the RTK known to promote angiogenesis. We, therefore, determined the effect of 194-A on intratumoral vasculature. We utilized a high frequency volumetric power Doppler ultrasound (HF-VPDU) to measure blood flow within large vessels of tumor vasculature with high flow velocities. Though HF-VPDU is a non-contrast-enhanced imaging, therapeutical effects can be recognized on the depicted vessels visually.  Figure 2(a) showed that the intratumoral vascularity in orthotopic grafts was drastically decreased compared with control tumors after the 10-day 194-A treatment. The antiangiogenic effect of 194-A was also verified by immunohistochemical analysis with an endothelial cell marker, CD31, on primary tumor tissue (Figure 2(b), upper panel). Administration of 194-A at 50 mg/kg p.o. produced 64% inhibition of the microvessel density (MVD) relative to vehicle-treated 4T1 tumors (Figure 2(b), bottom panel). *In vitro*, a dose-dependent decrease in VEGF-A-induced HUVECs proliferation was observed upon addition of 194-A (Figure 2(c)). 194-A at 1 *μ*M significantly inhibited VEGF-A-induced HUVECs proliferation. Similar inhibitory effect by 194-A on HUVECs migration was also observed (Figure 2(d)). Furthermore, we examined the effect of 194-A on VEGF-A-induced VEGFR-2 activity and their downstream signaling targets in primary HUVECs. 194-A at 1 *μ*M inhibited VEGF-A-induced phosphorylation of VEGFR-2 (Tyr 1054), ERK1/2, and Akt significantly (Figure 2(e)). Overall, these data demonstrate that administration of 194-A suppresses angiogenesis in 4T1 tumors, which may account for the antitumor activity of 194-A.

### 3.3. Significant Antimetastatic and Antilymphangiogenic Effect of 194-A in the 4T1 Tumor Model

**Figure 3:**
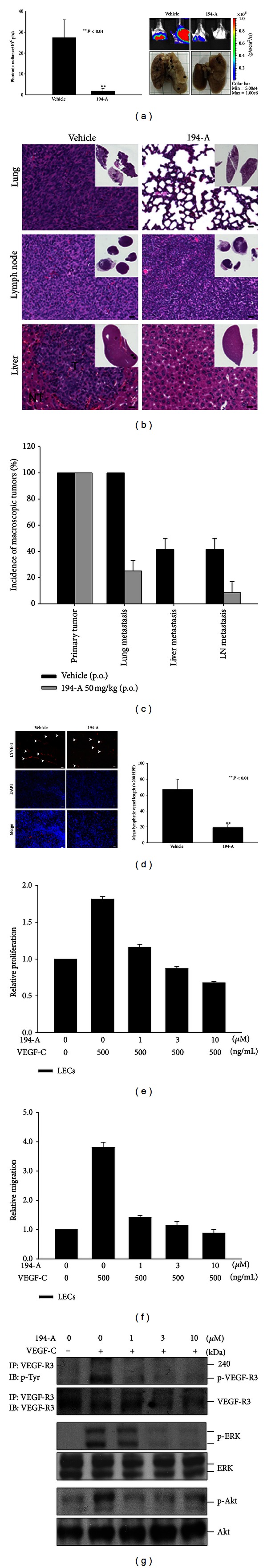
Significant antimetastatic and antilymphangiogenic effect of 194-A in the 4T1 orthotopic graft model. (a) 4T1-Luc^+^ (5 × 10^5^ cells/mouse) were orthotopically implanted into the fat pad of male BALB/c mice. Mice were p.o. administered with 50 mg/kg/day of 194-A or vehicle control for 30 days. At the end of the study, lung metastasis was measured by *in vivo* bioluminescent signals and *ex vivo* visualization. Arrow indicates metastatic tumor nodules. (b) Histological analyses of lung, liver, and lymph node metastasis from vehicle or 194-A-treated group. T, tumor part; NT, nontumor part. (c) The incidence of tumor metastasis was evaluated by histological analyses of the lung, liver, and lymph node. Mean values of two replicates;* bars*, SE. (d) Cryostat sections of 4T1 tumors, from mice treated with 194-A or vehicle control, were stained against LYVE-1 for the quantification of the mean lymphatic vessel length (LVL). Vessel length per 200x field was assessed from 3 to 4 fields per tumor section. *Columns*, mean (*n* = 6); *bars*, SE. **P < 0.01 as compared to the vehicle control group. Scale bar, 20 *μ*m. (e) and (f) Relative proliferation or migration of LECs grown in serum-free media supplemented with 500 ng/mL of VEGF-C as indicated. Proliferation (e) or migration (f) was reduced in a dose-dependent manner in response to 194-A treatment. Mean values of three replicates, normalized to the untreated controls; *bars*, SE. (g) 194-A inhibited VEGF-C-induced activation of VEGFR-3 and its common downstream signaling molecules. Serum-starved LECs were pretreated with 194-A for 30 min and then stimulated with 100 ng/mL VEGF-C for 10 min. Lysates were subjected to immunoprecipitation (IP) with VEGFR-3-specific antibody and resolved in SDS-PAGE, or resolved in SDS-PAGE directly and probed with specific antibodies against p-Tyr, VEGFR-3, p-ERK1/2, ERK1/2 p-Akt, and Akt.

In contrast to modest tumor growth inhibition, formation of spontaneous lung metastasis was dramatically prevented by 194-A (~94% inhibition) as measured by luciferase expression (Figure 3(a)). Visual comparison of mouse lungs showed marked growth of lung metastasis in vehicle-treated group, but few established invasive metastasis in 194-A-treated group (Figure 3(a)). In addition, H&E staining revealed a significant reduction in the incidence of lung, liver, and lymph node tumor metastasis in response to 194-A treatment (Figures 3(b) and 3(c)). Metastasis to lymph nodes occurred in 8.5% ± 3.5% and to the lung in 25% ± 8% after 194-A treatment, whereas metastasis to the lymph nodes and lung occurred in 41.5 ± 8.5% and 100%, respectively, in vehicle-treated mice. Notably, immunofluorescent analysis with a lymph endothelial cell marker, LYVE-1, showed that the mean lymphatic vessel length (MLVL) was decreased by 194-A (~70% inhibition) compared to vehicle treatment (Figure 3(d)).* In vitro*, 194-A dose dependently inhibited VEGF-C-induced LECs proliferation and migration (Figures 3(e) and 3(f)). In parallel, the activation of VEGFR-3 and its downstream signaling pathway induced by VEGF-C were inhibited by 194-A in a dose-dependent manner and almost abolished by 194-A at 1 *μ*M (Figure 3(g)). These experimental results demonstrated the antilymphangiogenesis efficacy of 194-A, which at least in part accounts for its antimetastatic effect.

### 3.4. 194-A Reduces the VEGF-C and FGF-2-Induced Invasive Effects of Mammary Carcinoma Cell Lines

**Figure 4:**
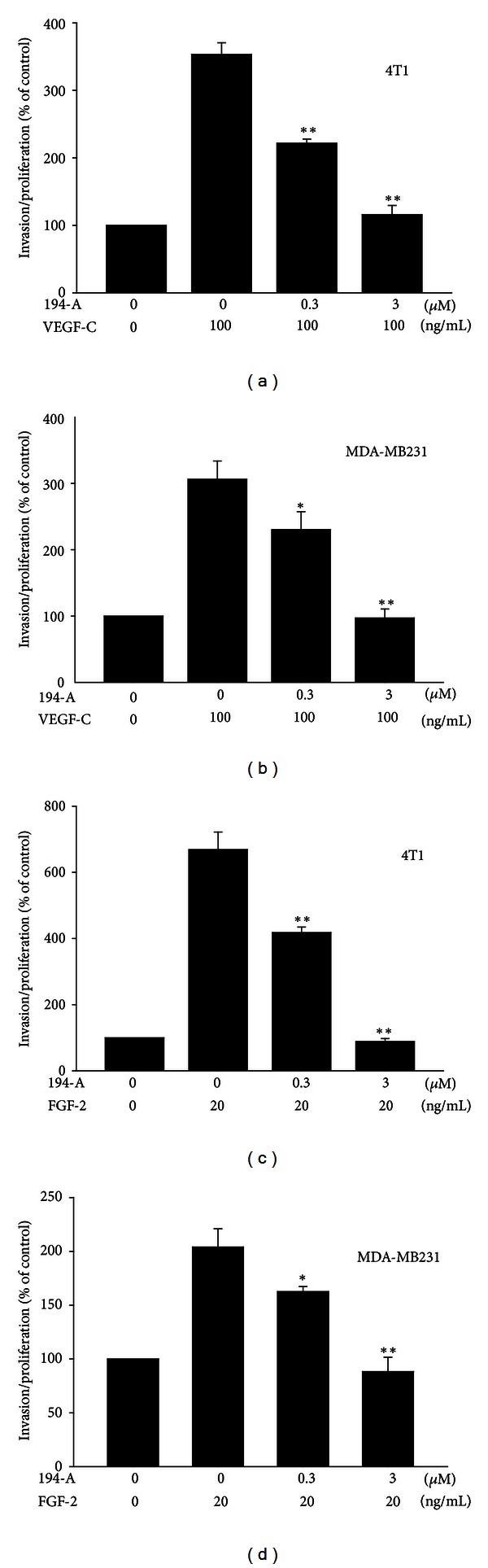
Effect of 194-A on VEGF-C or FGF-2-induced invasive effects in mammary carcinoma cells. Relative invasion of 4T1 and MDA-MB231 grown in low-serum (1% FBS) media supplemented with 100 ng/mL of VEGF-C (a) or 20 ng/mL FGF-2 (b) as indicated. Invasive ability was reduced in a dose-dependent manner in response to 194-A treatment. Mean value of three replicates, normalized to the untreated controls;* bars*, SE. *P < 0.05; **P < 0.01 as compared to the VEGF-C or FGF-2 treatment group.

Earlier studies of VEGFRs/FGFRs signaling on tumor cells have prompted us to evaluate whether 194-A would reduce the invasiveness of breast cancer cells [10, 20, 23, 29]. Here, we found that stimulation with VEGF-C in two VEGFR-3^+^ mammary carcinoma cell lines, 4T1 (Supplementary Figure 2(a)) and MDA-MB231 [10], resulted in a significant increase of invasive ability (Figures 4(a) and 4(b)). A dose-dependent decrease in VEGF-C-induced invasion was observed upon addition of 194-A (Figures 4(a) and 4(b)). Furthermore, increased levels of FGFR-1 and FGFR-2 expression were observed in 4T1 (Supplementary Figure 2(b)) and MDA-MB231 cells [30]. FGF-2 stimulation substantially increased the invasiveness of MDA-MB231 and 4T1 cells. Likewise, 3 *μ*M 194-A effectively suppressed the FGF-2 induced invasion of this two breast cancer cell lines (Figures 4(c) and 4(d)). These works further support the anti-metastatic potential of 194-A by targeting VEGFRs/FGFRs signaling.

### 3.5. The Antimetastatic Effects of 194-A Are Comparable with Sunitinib and Sorafenib

**Figure 5:**
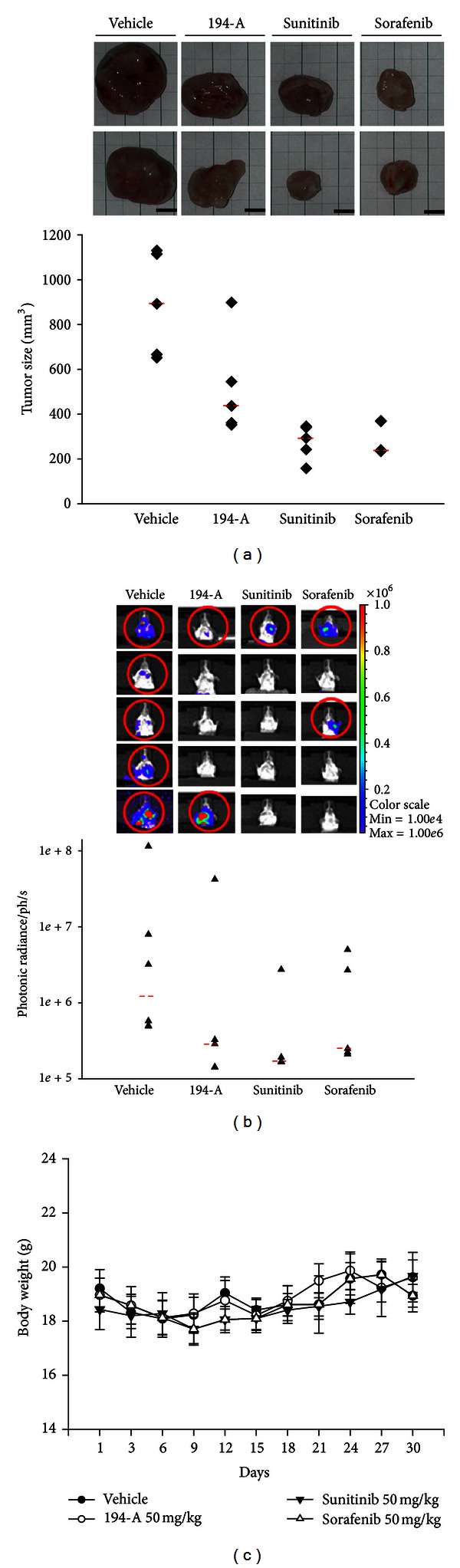
Comparison of the antitumor activity of 194-A with sunitinib or sorafenib in the 4T1 orthotopic graft model. (a) At the end of study, the tumor volume in mice treated with vehicle, 50 mg/kg/day 194-A (p.o.), sunitinib (p.o.), or sorafenib (p.o.) was measured by using a caliper. The median value of tumor volume was indicated. (b) Lung metastasis was measured at the end of study by *in vivo* bioluminescent signals. The median value of photon emissions was indicated.* Bars*, median values (*n* = 5). (c) 194-A, sunitinib, or sorafenib treatment did not affect the body weights significantly.* Lines*, mean (*n* = 5); *bars*, SE.

Given our observations that inhibition of VEGFRs/FGFRs signaling caused significant metastasis inhibition, we compared the therapeutic effects of 194-A, sunitinib, and sorafenib on metastasis of 4T1 to distant lung. Sunitinib and sorafenib are clinically used VEGFRs/PDGFR inhibitors with stronger potency than 194-A in inhibiting VEGFRs [31, 32]. We treated tumor-bearing mice using oral gavage with 50 mg/kg 194-A, sunitinib, or sorafenib daily starting 9 days after inoculation and administered for 30 days. While 194-A significantly reduced the primary tumor growth by 50% compared to the vehicle control (*P* = 0.047), sunitinib and sorafenib were shown to be more effective than 194-A (*P* = 0.009 and 0.048) (Figure 5(a)). Of note, using photon emissions detection of lung metastasis, we found that 194-A has comparable potency to sunitinib and sorafenib in preventing 4T1 metastasis to lung at the end of treatment (*P* = 0.75 and 0.92).  Figure 5(b) showed that the median value of photon emissions from the lungs was 2.9 × 10^5^ (for 194-A), 1.9 × 10^5^ (for sunitinib), 2.5 × 10^5^ (for sorafenib), and 1.4 × 10^6^ (for vehicle). No significant difference in body weight was detected among these four groups (Figure 5(c)). These experimental results suggest that the kinase inhibitory profile of 194-A (VEGFRs/FGFRs) might be feasible for preventing breast cancer metastasis.

## 4. Discussion

Much research has determined the requirement of angiogenesis for growth and progression of dormant lesions. It is of particular interest to determine whether antiangiogenic approach will not only reduce tumor growth but also block the progression of dormant lesions into aggressive cancers or metastasis in high-risk cancer patients. The multikinase inhibitor 194-A was an equally potent inhibitor of VEGFRs and FGFRs in cell-free assay (Supplementary Table 1). Administration of 194-A (p.o.) partially reduced tumor growth of 4T1 cells injected into the mammary fat pad and drastically reduced metastasis to distal organs. Histological analyses revealed decreased angiogenesis and lymphangiogenesis in tumor section from 194-A-treated mice, highlighting the impact of angiogenesis and lymphangiogenesis on tumor development and progression. 194-A treatment substantially reduced breast cancer cell motility and invasive ability associated with VEGFRs/FGFRs. The anti-metastatic potency of 194-A can be attributed by the synergistic inhibitory effects, which are achieved by targeting different signaling circuits, not only in endothelial cells, but also in cancer cells. Furthermore, we confirmed the effect of 194-A on tumor growth and metastasis using xenograft models of two highly metastatic cell lines, Lewis lung carcinoma, and B16/F10 (data not shown). These studies implicate the pivotal role of VEGFRs/FGFRs in cancer progression and metastasis.

Angiogenesis itself is a complex, multistep process that follows stage- and tissue-specific regulations. Various angiogenic factors have been identified that form an intimate network regulating angiogenesis. The most successful anti-angiogenic approaches are likely to involve combinatorial strategies to target the angiogenic factors appearing on the central stage of the angiogenesis network, such as VEGF, FGF, and PDGF. Some dual-action inhibitors have emerged that are more effective in restraining cancer growth. For example, sunitinib inhibits PDGF and VEGF receptors [33]; ZD6474 inhibits VEGFR and EGFR [34]; VX-322 inhibits FLT-3 and c-KIT [35]. Both VEGF and FGF are potent angiogenic factors. An intimate crosstalk exists among FGF-2 and the different members of the VEGF family during angiogenesis and lymphangiogenesis [36]. Previous reports have demonstrated that combinatory inhibition of VEGFR-1 and FGFR-1 produced an enhanced suppression of tumor growth in different types of cancer [37]. Our observation is in line with these studies, suggesting that blockade of both VEGFRs and FGFRs would efficiently inhibit angiogenesis.

Preventative antiangiogenic strategies could be especially useful in patients who are at high risk for developing metastasis. Few experimental studies in animals, as well as in clinical trials, have already shown promising results. For example, angiostatin and endostatin reduced the formation of metastasis in the murine Lewis lung carcinoma model [38, 39]. Additionally, regional lymph nodes are often the primary sites for metastasis, emphasizing the importance of the lymphatic system in metastatic process. Blocking the lymphangiogenic process has reduced metastasis to both the lymph node and distant organs [40]. Dual inhibition of both VEGFR-3 and VEGFR-2 appeared to be a better strategy to suppress tumor metastasis as both VEGFR-2 and VEGFR-3 are essentially involved in tumor angiogenesis and lymphangiogenesis. Supportive evidence from previous report showed that combination treatment using anti-VEGFR-2 and anti-VEGFR-3 antibodies more potently decreased lymph node and lung metastasis than each antibody treated alone [41]. The results of our study also highlight the significance of inhibition of VEGFR-3 and VEGFR-2 kinases in lymphatic and vascular endothelial cells, which reduced lymphangiogenesis and angiogenesis, resulted in inhibition of regional lymph node and distal organs metastasis. 

The invasive ability of tumor cell is a critical determinant of the metastatic phenotype of human cancers. Several sets of growth factors and their cognate receptors have been reported to be importantly involved in the regulation of tumor invasion and metastasis [42]. Earlier, we have demonstrated that the VEGF-C/VEGFR-3 signal directly promote cancer cells invasion and increase both lymph node and lung metastasis in xenograft model of human lung adenocarcinoma [10]. Treatment with a soluble form of VEGFR-3 (Flt4/Fc) suppressed lung and lymph node metastasis of two distinct lung tumor cell lines (A549 cells and VEGF-C-overexpressing H928 cells) [10]. 194-A might, therefore, directly inhibit VEGFR-3 in tumor cells, to down-regulate invasive activity and suppress tumor metastasis. Furthermore, it has also been demonstrated that FGFRs can mediate cell proliferation and the invasive ability of breast cancer cells [20, 29]. Indeed, both 4T1 (Supplementary Figure 2(b)) and MDA-MB-231 [30] cells expressed high levels of FGFR-2. We found that 194-A inhibited FGF-2-induced invasive ability of 4T1 cells more significantly than other chemoattractant (fibronectin) (data not shown). These observations suggest that the antitumor metastasis activity of 194-A *in vivo* might be, at least in part, attributed to reduced tumor cell invasiveness via inhibition of VEGFR-3- and FGFRs-mediated signaling on tumor cells.

Multiple pathways promote tumor angiogenesis and lymphangiogenesis. To restrict the metastatic spread of cancer, the ideal drug should counteract the essential angiogenic and lymphangiogenic factors produced by cancer cells and/or stromal cells during tumor progression. Clinically, VEGFRs/FGFRs expression is associated with poor prognosis in multiple cancer type. 

## 5. Conclusion

This study has validated the VEGFRs/FGFRs-mediated signaling pathways as a potential therapeutic target for inhibiting the metastatic spread of tumor cells. These encouraging data support further evaluation of VEGFRs/FGFRs inhibition for the treatment of highly metastatic cancer. It will also be tempting to determine whether this inhibition combined with conventional cytotoxic therapy could result in added efficacy without drawbacks in the near future. 

## Supplementary Material

Supplementary Table 1: Kinase selectivity profile of 194-A.
Supplementary Figure 1: Chemical structure of 0100194-A.
Supplementary Figure 2: The mRNA expression levels of VEGF-C, VEGF-R3,FGF-R1, FGF-R2, FGF-R3, and FGF-R4 in 4T1 cells.
