# Supplementary material for: Inhibition of Metastatic Potential in Breast Carcinoma In Vivo and In Vitro through Targeting VEGFRs and FGFRs
Source: Evid Based Complement Alternat Med. 2013 Jun 3;2013:718380. doi: 10.1155/2013/718380 (PMC3686112; doi:10.1155/2013/718380)
Supplement: Supplementary file 1 — Supplementary Table 1: Kinase selectivity profile of 194-A. Supplementary Figure 1: Chemical structure of 0100194-A. Supplementary Figure 2: The mRNA expression levels of VEGF-C, VEGF-R3,FGF-R1, FGF-R2, FGF-R3, and FGF-R4 in 4T1 cells. [file 718380.f1.pdf]

Supplemental Figure 1

0100194-A

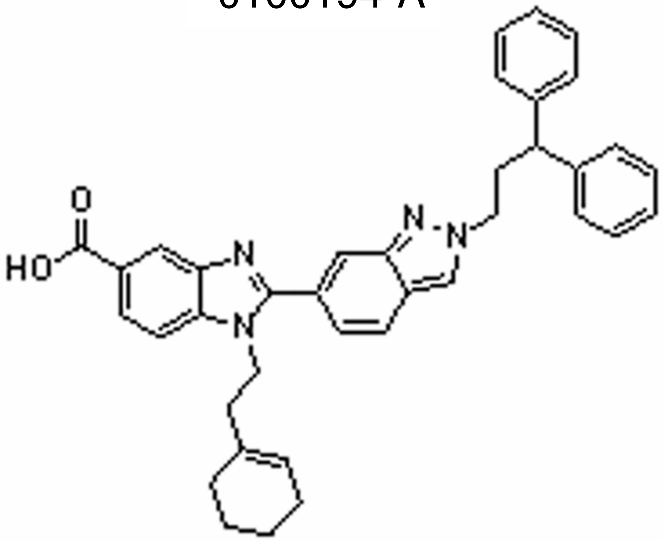

Supplemental Figure 1. Chemical structure of 0100194-A

Supplemental Figure 2

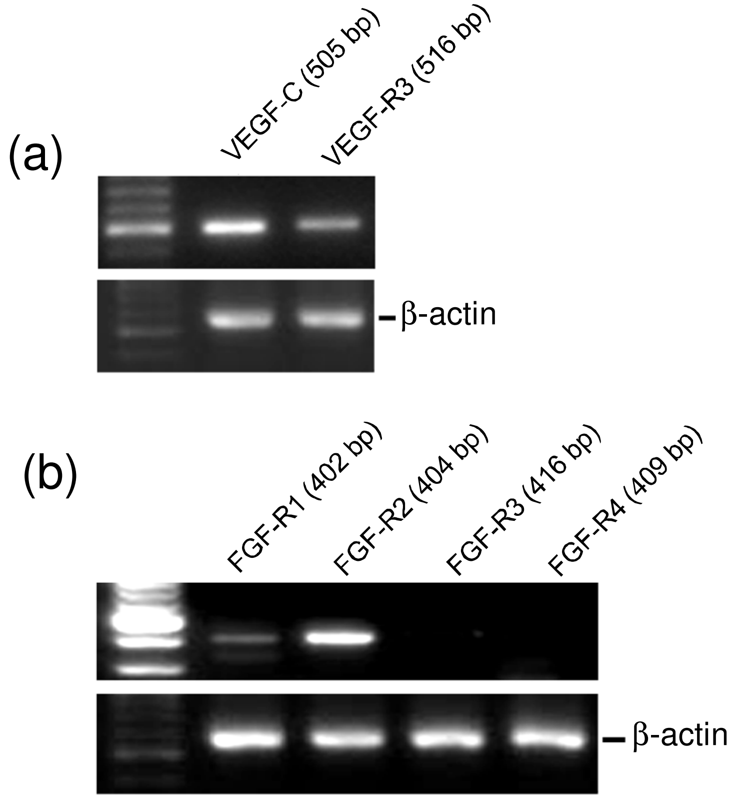

Supplemental Figure 2. mRNAs were isolated from 4T1-Luc$^+$ cells and amplified by using the following primers: VEGF-C: sense 5'-CCA CAG TGT CAG GCA GCT AA-3' and antisense 5'-TCC TGG ATC ACA ATG CTT CA-3'; VEGF-R3: sense 5'-GCC CTA CTG CAA GGT GCT AC-3' and antisense 5'-GCT TCT CTC CAA CCA ACA GC-3'; FGF-R1: sense 5'- TCA CAG CCA CTC TCT GCA CT-3' and antisense 5'- GGT TTG GTT TGG TGT TGT CC; FGF-R2: sense 5'-GAA CCA GAA GGA GCA CCG TA-3' and antisense 5'-CGC TGT AAA CCT TGC AGA CA-3'; FGF-R3: sense 5'-CCA CCG ACA AGG AGC TAG AG-3' and antisense 5'- AAC ATT GGC CAG AAC AGG AC-3'; FGF-R4: sense 5'- TGT CTA CCC ACA GCA AGC AC-3' and antisense 5'-GTC GCT GTA CAC CTT GCA GA-3'. The cycling conditions were 10 min polymerase activation at 95 °C followed by 32 cycles at 95 °C for 15 s, 58 °C for 60 s, and 72 °C for 60 s.

Supplemental Table 1

Kinase selectivity profile of 0100194-A

Kinases	0100194 IC_{50} (μM) (*n*=2)	Kinases	0100194 IC_{50} (μM) (*n*=2)
VEGF-R2	0.55	FGF-R3	>10
VEGF-R3	0.48	EGFR	>10
FGF-R1	1.33	PDGFRα	>10
FGF-R2	1.06	PDGFRβ	>10
FGF-R4	0.94	FAK	>10
HER2	9.4	ABL2	>10
SRC	7.58	IGF1R	>10

Supplemental Table 1. Kinase selectivity profile of 194-A. The kinase assays were performed by the MDS Pharma Services. Human VEGF-R2, VEGF-R3, PDGF-Rα, PDGF-Rβ, and FGF-R1~4 were expressed and purified from Sf21 cells. SRC, FAK, and HER2 were expressed and purified from Sf9 cells. EGFR was expressed and purified from A431 cells. γ-[^{P}32] ATP or ATP was used as the phosphate donor and poly (Glu:Tyr) peptide was used as the acceptor.
